# Detection and identification of *Chlamydia* spp. from pigeons in Iran by nested PCR and sequencing

**DOI:** 10.18502/ijm.v12i4.3937

**Published:** 2020-08

**Authors:** Nadia Golestani, Payam Haghighi Khoshkhoo, Hossein Hosseini, Gita Akbari Azad

**Affiliations:** Department of Clinical Sciences, School of Veterinary Medicine, Karaj Branch, Islamic Azad University, Karaj, Iran

**Keywords:** *Chlamydia psittaci*, Polymerase chain reaction, Pigeons, Genotype B, *Chlamydia avium*

## Abstract

**Background and Objectives:**

*Chlamydia psittaci*, an obligate intracellular, Gram-negative zoonotic pathogen, has eight serovars and nine genotypes isolated from avian species with higher frequency in parrots and pigeons. The aim of this study was to characterize *Chlamydia* spp. using nested PCR and sequencing.

**Materials and Methods:**

A total of 270 pharyngeal swab samples collected randomly from asymptomatic pigeons of 30 pigeon aviaries in Tehran province. DNA was extracted with specific kit and amplified by specific primers in the first PCR and outer membrane protein A *(ompA)* gene in the second PCR. Positive samples were sequenced and phylogenetic tree analyzed based on the *ompA* gene.

**Results:**

Records showed that 16 of 30 (53%) pigeon aviaries were positive for *Chlamydia* spp. Phylogenetic tree analysis revealed that 15 of 16 (93.7%) positive samples, belonged to *C. psittaci* genotype B whereas the other sample belonged to *C. avium. C. psittaci* detected in 50% of pigeon aviaries that is high rate in Tehran province.

**Conclusion:**

As *C. psittaci* is a zoonosis and life threaten pathogen for human being, these results indicate the significance of it detection in asymptomatic pigeons. Also, this is the first report of *Chlamydia avium* presence in Iranian pigeons which its zoonotic potential is still unknown.

## INTRODUCTION

Members of *Chlamydiaceae* family are obligate intracellular coccoid, Gram-negative bacteria which are transmitted by biologically inactive particles named elementary bodies (Ebs) (1). *Chlamydi psittaci* is the most common species which causes infection principally in parrots as psittacosis (ornithosis), pigeons (Columbiformes), doves and mynah birds. Affected birds can be asymptomatic; however, common clinical signs are weight loss, diarrhea, anorexia, polyuria, respiratory signs (dyspnea), conjunctivitis, hyperthermia, abnormal excretions, reduced egg production and sudden death (2). Recently, *Chlamydi avium* had identified as a new member of this family which causes respiratory disease and diarrhea in pigeons and psittacine (3). Several European studies reported this bacterium in pigeons (3–5), although the zoonotic ability of this bacterium is still an enigma. Psittacosis in humans has similar symptoms to influenza which can lead to pneumonia and non-respiratory health problems such as endocarditis, myocarditis, meningitis and conjunctivitis (6).

*C. psittaci* is a zoonotic pathogen with eight serovars and nine genotypes (A to F and E/B in avian). It has been detected from 467 avian species and 30 orders (7, 8). Genotype A and F are mainly detected from infected parrots, cockatoos, parakeets, genotype B in pigeons and genotypes C and D in ducks and turkeys respectively (9). Genotype E is isolated from a wide range of avian including turkeys, ducks, pigeons, ostriches and rheas, similarly, genotype E/B is associated with ducks, pigeons, and gray parrot (10). Also, provisional genotype I has been recently identified in Cockatiels (11).

All *C. psittaci* genotypes can be transmitted to humans or other mammals and cause psittacosis. This is mostly by direct contact with contaminated aerosol’s inhaling, eye secretions, feather dust, or dried faeces from an infected animal or environmental contamination by droppings and spreading by birds which are asymptomatic carriers (12). Firstly, Meyer (13) reported two psittacosis cases transmitted from feral pigeons to humans. Harkinezhad (14) suggested that pigeons and parrots cause more infection, using nested PCR and ELISA tests on 540 persons. Later on, several studies reported human psittacosis cases and identified *C. psittaci* genotype A, B or C in several cases with respiratory symptoms and conjunctivitis especially in individuals contacting with psittacine and pigeons in Belgium (15).

The prevalence of *C. psittaci* was measured in 11 European countries such as Switzerland, Slovenia, Spain, Italy, Germany, France, Bulgaria, Croatia, Bosnia and Herzegovina with an infection rate of 46% (16). *C. psittaci* infection rate has been recently detected in Iranian pigeons in some regions of Iran using molecular techniques. The last survey of *C. psittaci* average infection rate in Iranian pigeons was 18% which was conducted by Chaharmahal-va-Bakhtiari (17). Similarly, the detection rate of *C. psittaci* in birds including pigeons from the North-east of Iran was 18.5% (11). Another finding in Ahwaz indicated *C. psittaci* infection rate of 0.7% in asymptomatic pigeons (18).

As *C. psittaci* infection rate is high worldwide and all genotypes can threaten human’s health (16) the major problem is hard clinical diagnosis of this infection for similar symptoms to influenza and the high price of molecular techniques for special equipment and trained personnel. The current study was aimed to detect the prevalence of *C. psittaci* in pigeon aviaries at Tehran Province, Iran. This is run by conducting nested PCR and identify the genotype by phylogenetic tree based on *ompA* gene sequence precisely and helps to diagnose the prevalence of this pathogen for better treatment and prevention.

## MATERIALS AND METHODS

### Sampling.

In 2018, 30 pigeon aviaries were chosen randomly using blind sampling method in Tehran Province. These pigeon aviaries managed and owned by privates and each pigeon aviary had at least 200 pigeons with wide variety of pigeon’s breed. Nine pharyngeal swabs were obtained from each pigeon aviary with no specific clinical signs of infection. After pooling, a total number of 270 pharyngeal samples were collected from 30 pigeon aviaries.

### DNA extraction and nested PCR.

Template DNA was extracted using Cinapure DNA kit (CinaClon®, Tehran, Iran) according to manufactures’ protocol. Detection of *C. psittaci* was based on nested PCR technique by partial replication of the *ompA* gene, via two sets of specific primers (Table 1). Each set of primer was used for one stage. PCR reactions were done in 20 μl volume containing 2 μl of 10×PCR buffer, 1 μl 50 mM MgCl_2_, 0.5 μl of 1250 μM dNTPs, 1 μl of each forward and reverse primer, 1 U SmarTaq^TM^ DNA polymerase and 3 μl cDNA. The samples were put in a programmed thermocycler as following: initial detachment step at 95°C for 5 minutes, followed by 35 cycles of 10 seconds at 95°C, 10 seconds at 56°C 10 seconds at 72°C, and a final extension step at 72°C for 5 minutes. The product produced from the first round underwent for the second run similar to the first run mixture (and cycling program) with internal primers (Table 1).

**Table 1.** Primers Sequence (*OmpA* gene)

**Nested PCR**	**Primers**	**Sequence (5′-3′)**	**Reference**
First round	191CHOMP	GCI YTI TGG GAR TGY GGI TGY GCI AC	(19)
	CHOMP371	TTA GAA ICK GAA TTG IGC RTT IAY GTG IGC IGC	(19)
Second round	218PSITT	GTA ATT TCI AGC CCA GCA CAA TTY GTG	(19)
	CHOMP336s	CCR CAA GMT TTT CTR GAY TTC AWY TTG TTR AT	(20)

### Gel electrophoresis.

PCR amplicons were electrophoresed on 2% Agarose gel (UltraPure Agarose Invitrogen) and visualized by Ethidium-Bromide staining under ultra-violet (UV) Transilluminator (Vilvent, France). Specific band at 389 to 404 bp was considered positive for *C. psittaci*.

### Sequence analysis.

Sixteen positive isolates (one isolate per each positive pigeon aviary) were selected and the PCR product was purified using High Pure PCR Product Purification Kit (Roche Life Science, Germany) and sent to Bioneer Laboratory (South Korea) for sequencing (21). The sequences were compared to the reference sequences of *Chlamydia* in GenBank (NCBI). The phylogenetic tree were arranged by Mega Software Version 7 by the neighbor joining method based on Kimura 2-parameter model with 1000 bootstrap replicates (22). The accession numbers of 16 *ompA* gene of *Chlamydia* spp. introduced to GenBank with the following accession numbers:

*C._ psittaaci*_H3120-1/19, *C._ psittaaci*_H3120-3/19, *C._ psittaaci*_H3120-4/19, *C._ psittaaci*_H3120-7/19, *C._ psittaci* H3120-8/19, *C._ psittaci* H3120-9/19, *C._ psittaci* H3120-10/19, *C._ psittaci* H3120-12/19, *C._ psittaci* H3120-13/19, *C._ psittaci* H3120-17/19, *C._ psittaci* H3120-18/19, *C. psittaci* H3120-21/19, *C._ psittaci* H3120-23/19, *C._ psittaci* H3120-24/19, *C._ psittaci* H3120-28/19, *C. _avium* H3120-30/19.

## RESULTS

### Detection of *Chlamydia*.

Chlamydial DNA was detected in 16 of 30 pigeon aviaries (53%) after visulaizing specific DNA band with 404 bp length on 2% agarose gel. Thus, results indicate the high rate of detection for this pathogen. No symptoms of chlamydiosis were recorded in positive pigeon aviaries.

### Phylogenetic analysis and genotyping.

**Fig. 1.**
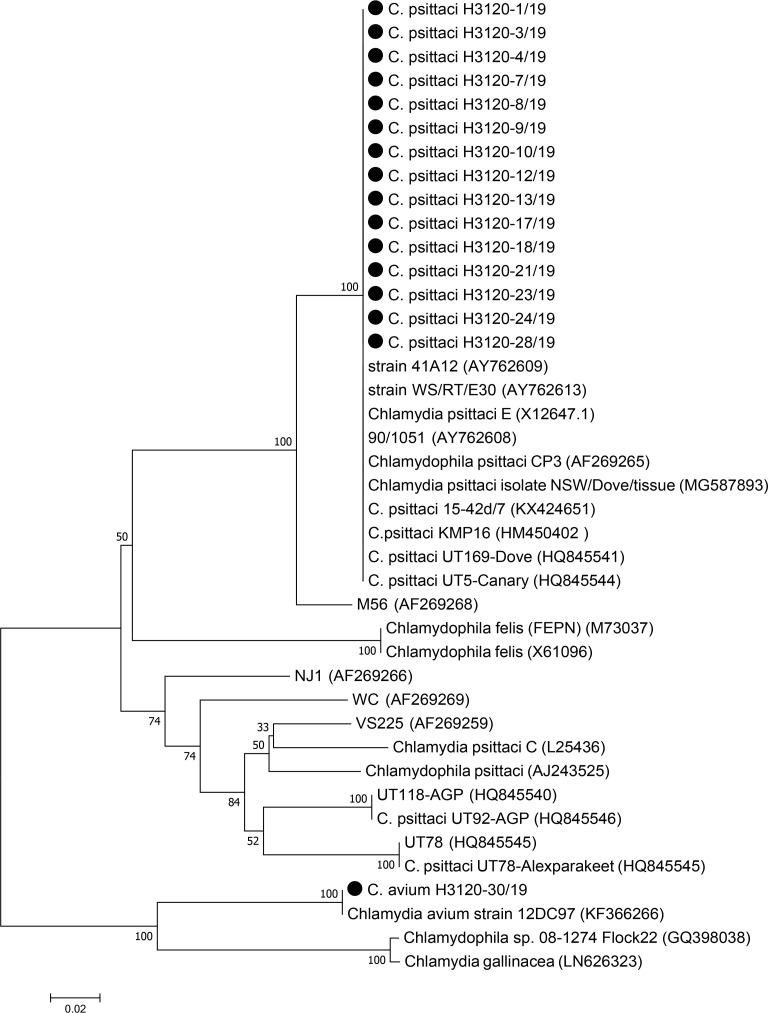
Phylogenetic tree based on gene *ompA* sequence of 16 samples from pigeons in Tehran Province

16 samples from positive Chlamydia DNA aviaries (one from each) selected for *ompA* gene genotyping. Based on *ompA* sequence analysis and comparing the results sequences with the reference samples (Table 2) in GenBank, 15 of 16 samples were classified as *C. psittaci* genotype B (Fig. 1). Nucleotide sequences of the 15 positive samples had 100% sequence identity with each other. Thus, Table 2 lists one positive sample (representative of 15 positive samples) that showed 100% nucleotide similarity to *C. psittaci* strains UT169-Dove (accession number HQ845541) and UT5-Canary (accession number HQ84554). Moreover, these *C. psittaci* strains were highly homologous with *C. psittaci* isolate NSW/Dove/tissue (accession number MG587893) (Fig. 1).

**Table 2.** Indicates nucleotide similarities of isolated sequences in this study with several standard reference sequences of *Chlamydia* spp. *C. psittaci* strain H3120-1/19 and *C. avium* H3120-30/19 are indicators of our positive samples.

**No**	**Species**	**Genotype**	**Strain Name**	**Accession No**	**Number of Nucleotide similarities (%)**													

					1	2	3	4	5	6	7	8	9	10	11	12	13	14
1	*C. psittaci*	B	H3120-1/19															
2	*C. psittaci*	B	UT5-Canary	HQ84554	100													
3	*C. psittaci*	B	NSW/Dove/tissue	MG587893	100	100												
4	*C. avium*	_	H3120-30/19		77.7	77.7	77.7											
5	*C. psittaci*	M56	_	AF269268	96.8	96.8	96.8	77.7										
6	*C. psittaci*	F	VS225	AF269259	85.1	85.1	85.1	77.7	87.2									
7	*C. psittaci*	C	_	L25436	86.2	86.2	86.2	76.6	87.2	91.5								
8	*C. psittaci*	D	NJ1	AF269266	88.3	88.3	88.3	76.6	89.4	89.4	92.6							
9	*C. psittaci*	WC	_	AF269269	88.3	88.3	88.3	77.7	90.4	89.4	87.2	92.6						
10	*C. gallinacea*	_	_	LN626323	73.4	73.4	73.4	89.4	74.5	75.5	77.7	78.7	74.5					
11	*Chlamydophila felis*	_	_	X61096	89.4	89.4	89.4	77.7	90.4	87.2	89.4	90.4	89.4	76.6				
12	*Chlamydophila psittaci*	_	_	AJ243525	87.2	87.2	87.2	78.7	89.4	95.7	92.6	91.5	92.6	76.6	90.4			
13	*C. psittaci*	Provisional I	UT92	HQ845546	85.1	85.1	85.1	76.6	87.2	93.6	88.3	87.2	90.4	76.6	87.2	95.7		
14	*C. psittaci*	Provisional J	UT78	HQ845545	88.3	88.3	88.3	80.9	90.4	94.7	91.5	90.4	92.6	77.7	89.4	97.9	95.7	
15	*C. avium*	_	12DC97	KF366266	77.7	77.7	77.7	100	77.7	77.7	76.6	76.6	77.7	78.7	89.4	77.7	76.6	80.9

In this investigation, *ompA* phylogram identified *C. avium* in 1 out of 16 samples. This is the case that for the first time is reported for pigeons in Iran. This nucleotide sequence had 100% similarity to *C. avium* 12DC97 (accession number KF366266). This nucleotide sequence (*C. avium* H3120-30/19) had 89.4% similarity with *C. gallinacea* and 77.7% homology with other 15 sequenced samples known as *C. psittaci* genotype B (Table 2).

## DISCUSSION

Chlamydiosis is a notable systemic disease in birds which can cause similar symptoms to influenza in mammals and humans (23). Consequently, *C. psittaci* detection in pigeons in Tehran Province, Iran improves better understanding of this epidemic infection in the birds and humans.

In this study, it was found that prevalence of *C. psittaci* in pigeons in Tehran Province is 50%. The frequency of this pathogen in this study was almost higher than other studies in Iran (11) (17). The reason for this issue is attributed to high-frequency trading of pet birds from other regions of Iran to Tehran for higher price due to higher demands in the capital, Tehran.

This work is the first experimental study on detecting the prevalence of *C. psittaci* in pigeons of Tehran province. The studies conducted in the southwestern part of Iran, showed *C. psittaci* infection in pigeons using molecular techniques. Mahzonieh (24) confirmed *C. psittaci* infection rate of about 52% in pigeons in Chaharmahal Bakhtiari in Iran by nested PCR technique and recently they confirmed the average infection rate of 18% (17). Khodadadi et al. detected the average infection rate of 13% in blood, liver, and muscle tissue of pigeons by the same method in the same region (25). Their results indicated a significant decrease of this infection for pigeons in this province. Furthermore, Ghorbanpoor (18) showed *C. psittaci* detection rate of 0.7% with PCR by analyzing *pmp* genes, 16s and 23s rRNA intergenic space in asymptotic pigeons of Ahwaz, south west of Iran. A recent study focused on the North region of Iran confirmed the infection rate is about 18% in asymptomatic and symptomatic birds including pigeons by nested PCR (11). Analysis and comparison of the above studies revealed that detection of *C. psittaci* from pharyngeal and fecal samples in pigeons with PCR technique is a proper method. Chlamydiosis in birds rose significantly in the Europe in recent years and their reports on chlamydia infections European countries including Switzerland (5), Germany (26), Belgium (27) and Spain (28). Prevalence of *C. psittaci* in pigeons confirmed about 16%, 29%, 40% and 52% respectively.

*C. psittaci* genotype B is endemic and particularly associated with European pigeons (16, 21). Madani confirmed the presence of *C. psittaci* genotype B in Dove and Canary in Iran by partial sequencing of *ompA* (29). The more recently Abbasi et al. (11) found *C. psittaci* genotype B in symptomatic pigeons by partial sequencing of *ompA* in the North of Iran. Several studies identified *C. psittaci* genotype B in pigeons by PCR in different countries including the Netherlands (4), Belgium, Germany and Italy (30).

*OmpA* sequenced revealed *C. avium* in one pigeon aviary in Tehran province for the first time. Firstly, Sachse et al. (3) identified *C. avium* and *C. gallinacea* as a member of *Chlamydiaceae* which endangers pigeons and psittacine in Germany. Similarly, Sariya et al. (31) used nested PCR technique for chlamydia detection in pigeons and identified one of the samples related to *C. avium* by sequencing of *ompA*. This is the same as our results when the majority samples were grouped under genotype B in pigeons. The recent experiments found whole genomic sequences of *C. avium* isolated from pigeons in Italy (32), the Netherlands (4) and Switzerland (5) by PCR. Further experiments should be done to determine the zoonotic ability and the virulence of this strain.

According to our findings, *C. psittaci* was the most common species found in all experiments and the prevalence of this infection in pigeon aviaries is considerably high. Due to the high density of pigeons in urban and rural areas, dust inhalation and daily unprotected contacts of pet birds with owners and other involved people such as breeders, veterinarians, laboratory and farmworkers is a hazard for the public health (33). As all *C. psittaci* genotypes can infect humans, characterization of this pathogen is recommended, although *C. psittaci* genotype B and E causes a less virulent infection (2). There is no experiment in *C. psittaci* genotyping in Iranian people contacting birds. The last survey in Iran identified *C. psittaci* on humans which was not possible due to insufficient DNA (17).

Besides the zoonotic potential for high-risk people such as children, elders and people under immuno-suppression conditions, the risk of infection for pet birds and poultry is high for all mammals and humans. Psittacosis can be treated by tetracycline or macrolides; however the antibiotic resistance in pet birds is reported frequently for the difficult diagnosis of this infection and similar symptoms, therefore the regular use of prophylactic antibiotics is not recommended (34).

In conclusion, this study indicates a significant number of infected pigeon aviaries by *C. psittaci* in Tehran province. As *C. psittaci* is a zoonotic pathogen, biosecurity, diagnostic methods and therapeutic principles plus monitoring and reporting have major role in reducing the *C. psittaci* transmission. Moreover, further experiments should address combination and multiple bacterial infection especially *C. avium* to identify possible synergies or competitive effects between individual factors.
